# Test-retest reliability and measurement error of the Danish WHO-5 Well-being Index in outpatients with epilepsy

**DOI:** 10.1186/s12955-018-1001-0

**Published:** 2018-09-06

**Authors:** Liv Marit Valen Schougaard, Annette de Thurah, Per Bech, Niels Henrik Hjollund, David Høyrup Christiansen

**Affiliations:** 1 AmbuFlex/WestChronic, Occupational Medicine, Regional Hospital West Jutland, University Research Clinic, Aarhus University, Herning, Denmark; 2 Department of Rheumatology, Aarhus University Hospital, Aarhus, Denmark; 3 Department of Clinical Medicine, Aarhus University, Aarhus, Denmark; 4 Psychiatric Research Unit, Psychiatric Centre North Zealand, University of Copenhagen, Hillerød, Denmark; 5 Department of Clinical Epidemiology, Aarhus University Hospital, Aarhus, Denmark; 6 Department of Occupational Medicine, Regional Hospital West Jutland, University Research Clinic, Herning, Denmark

**Keywords:** Patient reported outcome measures, Validation studies as topic, Reproducibility of results

## Abstract

**Background:**

The generic questionnaire WHO-5 Well-being Index (WHO-5), which measures the construct of mental well-being has been widely used in several populations across countries. The questionnaire has demonstrated sufficient psychometric properties; however, the test- retest reliability of the WHO-5 scale has yet to be determined. The aim of this study was to evaluate the test-retest reliability and measurement error of the Danish WHO-5 Well-being Index for outpatients with epilepsy. A further aim was to evaluate whether the method of administration (web, paper, or a mixture of the two modalities) influenced the results.

**Methods:**

Epilepsy outpatients aged ≥15 years from three outpatient clinics in Central Denmark Region were included from August 2016 to April 2017. The participants were randomly divided into four test-retest groups: web-web, paper-paper, web-paper, and paper-web. Test-retest reliability was assessed by intraclass correlation coefficients (ICC) and measurement error by calculating minimal detectable change (MDC) on the basis of the standard error of the measurement.

**Results:**

A total of 554 patients completed the questionnaire at two time points. The median duration between test-retest was 22 days. The pooled test-retest reliability estimate was ICC 0.81 (95% CI 0.78; 0.84). The estimated MDC was 23.60 points (95% CI 22.27; 25.10). These estimates showed little variation across administration methods.

**Conclusions:**

WHO-5 showed acceptable test-retest reliability in a Danish epilepsy outpatient population across different method of administration; however, the relatively large measurement error should be taken into account when evaluating changes in WHO-5 scores over time. Further research should be done to explore these findings.

## Introduction

Several considerations are important when selecting patient-reported outcome (PRO) measures for use in clinical practice. A PRO measure should be relevant to patients and clinicians and possess an adequate level of psychometric evidence for the instrument in the target population [1]. In Central Denmark Region, PRO measures have been used as the basis for follow-up in three epilepsy outpatient clinics since 2012 [2, 3]. Patients complete a web or paper-based questionnaire at home instead of having pre-scheduled appointments. Clinical resources could then be directed towards patients with actual need, and clinicians could use patients’ self-reported information to identify otherwise undetected problems. As depression is common in patients with epilepsy [4], valid and reliable measurement tools are necessary to identify relevant symptoms. For this purpose, the WHO-5 Well-being Index (WHO-5) was selected and has been used since 2012 for outpatients with epilepsy in Central Denmark Region.

WHO-5 is a generic unidimensional questionnaire reflecting the construct mental well-being during the last 2 weeks [5]. The scale was developed in 1998 and has been widely used [6]. WHO-5 includes five positive wording statements rated on a 6-point ordinal scale ranging from 5 “all of the time” to 0 “at no time”. Raw scores, which range from 0 to 25, are multiplied by 4 to obtain a percentage score ranging from 0 (worst) to 100 (best). A percentage score below 50 indicates poor mental well-being and a risk of depression. The WHO-5 has demonstrated sufficient psychometric properties in terms of construct validity, predictive validity, and internal consistency reliability in several patient populations including epilepsy [6–14]; however, the test- retest reliability of the WHO-5 scale has yet to be determined. Furthermore, few studies have explored the impact on consistency of using different methods of administration [15, 16].

The study aim was to evaluate the test-retest reliability and measurement error of the Danish WHO-5 Well-being Index for outpatients with epilepsy. A further aim was to evaluate whether the method of administration (web, paper, or a mixture of the two modalities) influenced the results.

## Methods

### Study population and setting

Patients with epilepsy aged ≥15 years from three outpatient clinics in Central Denmark Region were included from August 2016 to April 2017. The patients completed the questionnaire at two time points. First, they completed a questionnaire from the outpatient clinic based on their preferred web or paper administration method (test 1). Subsequently, approximately 2 weeks later, a letter was sent to the patients asking them to complete the same questionnaire again (test 2). The patients were randomly divided into four test-retest groups based on the method of administration at test 1 and test 2: web-web, paper-paper, web-paper, and paper-web. Three reminders were sent in test 1, but no reminders were sent to non-responders in test 2. The WHO-5 Well-being Index was included in the questionnaire in test 1. In addition, the questionnaire included other items, regarding, for example, seizures, symptoms, and general health. The general health construct was measured by using two items from the Danish version of the Short Form 36 Health Survey [17, 18]. A long interval between test administrations increases the risk of change in patients’ health status in a test-retest study, whereas a short interval increases the risk of recall bias [19]. The questionnaire in test 1 was sent to the patients as part of routine outpatient follow-up. Patients’ mental health was assumed to be stable during the time period from test 1 to test 2, since the health status of epilepsy patients is not likely to change over a period of 2 weeks. The patients were not asked in test 2 whether their mental health had changed within the time period.

### Data analyses

Descriptive statistics were generated for patient characteristics and for each item to determine the extent of missing values and floor- or ceiling effects, which were considered present if more than 15% had a score at the lower or upper end of the scale [19]. Cronbach’s alpha was used to assess internal consistency. The 95% confidence interval (CI) of the Cronbach’s alpha values was estimated by using the bootstrap method (1000 replications). The time interval between test 1 and 2 was calculated as the difference in number of days from the dates of responses. Test-retest reliability of the scale was assessed by intraclass correlation coefficients (ICC) agreement model 2.1 [20], with 95% CI, and for single items, kappa with squared weights and 95% CI was used. An ICC value of 0.70 is considered acceptable for group level analysis, but when evaluating individual patients, an ICC of 0.90 is recommended [19]. The kappa values were interpreted as following: < 0.2 (slight), 0.21–0.4 (fair), 0.41–0.6 (moderate), 0.61–0.8 (substantial), and 0.81–1.0 (almost perfect) [21]. Measurement error was assessed with differences between test 1 and 2 plotted against the means of the two measurements by Bland–Altman plots with 95% CI and 95% limits of agreement (LOA). LOA equals the mean change in scores between test 1 and 2 (mean change ±1.96 x standard deviation of the changes) and gives an indication of how much two scores can vary in stable patients. LOA are expressed in the units of measurement instrument and give a direct indication of the size of the measurement error [19]. The measurement errors reflect the within intraindividual variation and were estimated as the standard error of the measurement (SEM) [22]. SEM equals the square root of the error variance. The interpretation of a SEM estimate is not straight forward; therefore the SEM was converted into the minimally detectable change (MDC). MDC^95^ equals 1.96 ± √2 x SEM and indicates the smallest within-person change that can be interpreted as a “real” individual change above the measurement error [22]. Thus, a change in scores within the LOA or smaller than MDC^95^can be attributed to measurement error [19]. Patients with missing item values were excluded from the analyses. Two sensitivity analyses were performed to investigate whether the length of the time interval between test 1 and test 2 affected our results. In the first analysis, patients were excluded if the time period between test 1 and test 2 was above 30 days, and in the second analysis all patients with a time interval above 14 days were excluded. STATA 15 software (Stata Corp, College Station) were used for all statistical analyses.

## Results

### Patient and item characteristics

A total of 554/1640 (34%) patients responded to the questionnaire twice. The median age was 57.3 years (Table 1). The response-rates in the four test-retest groups ranged from 48% (web-paper and paper-paper) to 34% (web-web) to 9% (paper-web). Non-responders were more likely younger, paper-responders, and had lower self-reported general health in test 1 (data not shown). The median response time between test-retest was 22 days (inter quartile range 10 days). A total of 14 patients had missing values for WHO-5 in test 1 or 2 and were excluded from the analyses. Percentages of missing values ranged from 0.2 to 1.1%, and there was a tendency towards ceiling effects in all items (Table 2). Cronbach’s alpha was 0.89 (95% CI 0.87; 0.90) in test 1 and 0.89 (95% CI 0.87; 0.91) in test 2.

**Table 1:** Patient characteristics at time of test 1 among outpatients with epilepsy, *N* = 554

Gender, *n* (%) Male	286	(52)
Age, *y,* median (IQR)	57.3	(25.1)
Outpatient clinic, *n* (%)		
Aarhus	409	(74)
Holstebro	115	(21)
Viborg	30	(5)
General health^a^, *n* (%)		
Excellent	67	(12.1)
Very good	191	(34.5)
Good	209	(37.7)
Fair	68	(12.3)
Poor	19	(3.4)
WHO-5 score in test 1		
Median (IQR)	76	(24)
Mean (SD)	70.6	(19.5)
Missing, *n* (%)	5	(0.9)
WHO-5 score in test 2		
Median (IQR)	76	(24)
Mean (SD)	70.5	(19.2)
Missing, *n* (%)	9	(1.6)

^a^Item GH-1 from Short Form 36 Health Survey [17]

*Abbreviations IQR* inter quartile range, *SD* Standard deviation

**Table 2:** Item level distribution and weighted kappa of the WHO-5 Well-being Index (*N* = 554)

Item	Distribution (%) of the response options^a^									Test-retest Weighted kappa
	Item content		Missing	0	1	2	3	4	5	
1	I have felt cheerful and in good spirits	Test 1	0.2	0.5	5.2	5.6	12.3	61.6	14.6	0.70 (0.64; 0.76)
		Test 2	0.7	0.5	3.4	6.3	14.1	61.4	13.5	
2	I have felt calm and relaxed	Test 1	0.5	1.4	4.9	6.0	15.2	52.0	20.0	0.67 (0.59; 0.74)
		Test 2	0.5	1.3	2.7	7.9	13.4	56.9	17.3	
3	I have felt active and vigorous	Test 1	0.2	3.1	8.5	13.0	21.7	37.7	15.9	0.70 (0.65; 0.76)
		Test 2	0.5	2.9	9.0	13.5	19.7	41.9	12.5	
4	I woke up feeling fresh and rested	Test 1	0.2	4.7	10.6	10.5	18.8	40.4	14.8	0.72 (0.66; 0.77)
		Test 2	1.1	5.4	9.0	11.4	17.0	41.9	14.3	
5	My daily life has been filled with things that interest me	Test 1	0.5	0.9	5.4	7.0	16.6	51.3	18.2	0.68 (0.62; 0.74)
		Test 2	1.1	0.7	7.6	6.5	15.0	51.3	17.9	

^a^0 = At no time, 1 = Some of the time, 2 = Less than half of the time, 3 = More than half of the time, 4 = Most of the time, 5 = All of the time

### Test-retest reliability and measurement error of WHO-5

**Fig. 1:**
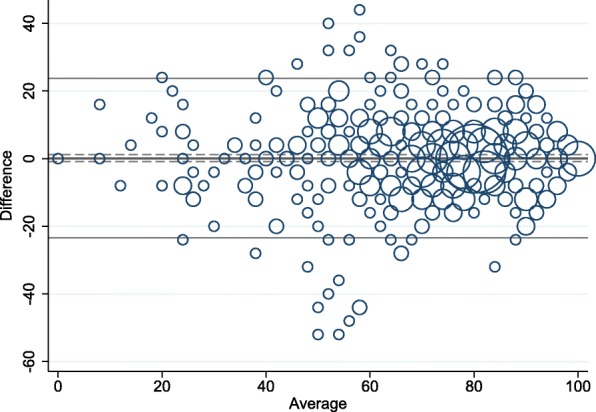
Differences in the WHO-5 Well-being Index score between test 1 and test 2 plotted against the mean, *N* = 540

**Fig. 2:**
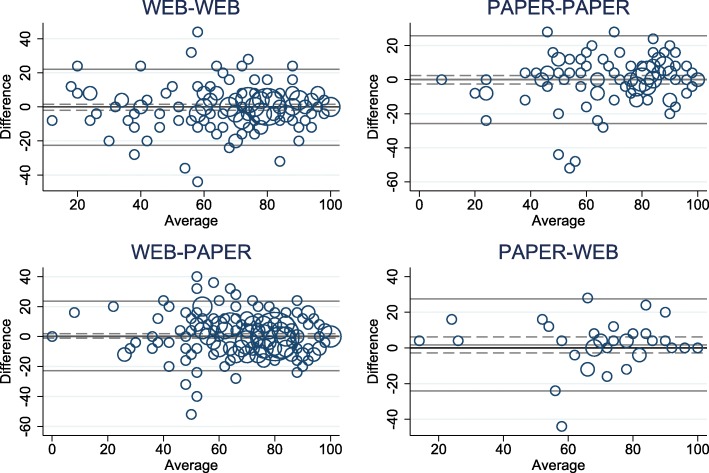
Differences in the WHO-5 Well-being Index score between test 1 and test 2 plotted against the mean in the four test-retest groups: web-web (*n* = 164), paper-paper (*n* = 107), web-paper (*n* = 233), and paper-web (*n* = 36)

Kappa values for the five single items were substantial (Table 2) [21]. The ICC of the pooled WHO-5 score was 0.81 (95% CI 0.78; 0.84) (Table 3). Differences between test 1 and test 2 plotted against the mean of the two tests with upper and lower LOAs are shown in Fig. 1. The estimated SEM was 8.51 points (95% CI 8.03; 9.05), which resulted in a MDC^95^ of 23.60 points (95% CI 22.27; 25.10). The analysis was repeated in the four test-retest groups (Table 3 and Fig. 2). Administration methods did not noticeably alter the estimates. The overall results did not change, when the analyses were repeated with restricted intervals between test 1 and 2.

**Table 3:** Test-retest reliability and measurement error for the WHO-5 Well-being Index between test 1 and test 2

WHO-5	N	Mean, (95% CI) Test 1	Mean (95% CI) Test 2	Difference (95% CI)	SEM (95% CI)	ICC (95% CI)	MDC^95^ (95% CI)
Pooled	540	70.58 (68.94; 72.21)	70.40 (68.78; 72.02)	0.18 (−0.84; 1.20)	8.51 (8.03; 9.05)	0.81 (0.78; 0.84)	23.60 (22.27; 25.10)
Web-web	164	69.83 (66.73; 72.93)	70.10 (67.01; 73.18)	−0.27 (−2.02; 1.49)	8.05 (7.26; 9.03)	0.84 (0.80; 0.89)	22.31 (20.13; 25.03)
Paper-paper	107	70.65 (66.41; 74.90)	70.69 (66.87; 74.51)	−0.04 (− 2.56; 2.49)	9.31 (8.21; 10.76)	0.81 (0.74; 0.87)	25.81 (22.76; 29.82)
Web-paper	233	71.10 (68.85; 73.33)	70.73 (68.37; 73.09)	0.36 (−1.17; 1.89)	8.36 (7.66; 9.20)	0.78 (0.73; 0.83)	23.18 (21.24; 25.49)
Paper-web	36	70.44 (63.63; 77.26)	68.78 (62.05; 75.50)	1.67 (−2.79; 6.12)	9.30 (7.55; 12.14)	0.78 (0.66; 0.91)	25.79 (20.92; 33.64)

*Abbreviations*: *WHO*-5 *WHO*-5 Well-being Index, *N* Number, *CI* Confidence Interval, *SEM* Standard error of the measurement, *ICC* Intra class correlation coefficient, *MDC* Minimal detectable change

## Discussion

Test-retest reliability of the Danish WHO-5 Well-being Index was found to be acceptable in an epilepsy outpatient population, but a relatively large measurement error was observed. The estimated MDC^95^ was 23.60 points, indicating that changes in the WHO-5 instrument must be substantial to ensure that a ‘real’ change is not due to measurement error. Methods of administration did not markedly influence the results.

This study follows the COSMIN framework [23, 24] and supplements earlier established psychometric properties of the WHO-5. Since we were unable to identify other test-retest studies of the scale, we believe this is the first study to determine the test-retest reliability of the WHO-5. Several studies have explored another aspect of reliability: internal consistency [8–14]. The Cronbach’s alpha of the WHO-5 in these studies ranged from 0.82 to 0.95, which is consistent with the findings in this study. However, this aspect determines the correlation between items within a scale and not the degree of agreement for repeated measurements over time [22, 24]. The unidimensionality of the WHO-5 scale has been confirmed by using Rasch item response theory analyses in both a younger and elderly population [14, 25].

Test-retest reliability should be assessed in a stable population with an appropriate time interval between measurements [22]. We assumed that the epilepsy outpatient population was stable and allowed a longer time interval. Sensitivity analyses were used to assess potential change in health status; however, excluding participants with longer intervals between test 1 and 2 did not substantially alter the estimates. Still, we cannot rule out that a change in patients’ health status had occurred and that this might have affected the ICC and measurement error estimates of the WHO-5 scale, as we did not collect information on the change in patients’ mental health status from test 1 to test 2.

The WHO-5 scale ranges from 0 to 100, and an MDC of 23.6 points observed in this study may indicate that longitudinal differences of at least 24 points are needed to detect a “true” within-person change. The relatively large measurement error observed in this study may be taken into consideration by researchers planning future clinical trials and clinicians who use the scale on the individual level in clinical practice to evaluate change over time. Furthermore, the tendency towards ceiling effect may produce difficulties in measuring longitudinal changes. Web, paper, or a mixture of the two modalities showed nearly the same test-retest reliability, which is consistent with other test-retest studies [15, 16].

One important limitation of this study is the possibility of selection bias. A very low response rate was observed especially in the paper-web group (9%). This may be due to the pragmatic design, which allowed patients to choose administration method for their response to test 1. In the Danish general population, a mean WHO-5 score of 70 points has been reported [26, 27]. This is comparable with the result in this study; however, the responders tended to be a healthier group of patients compared to non-responders in test 2 who had lower self-reported general health and mental well-being in test 1. The reliability estimates indicate how well patients can be distinguished from each other despite the presence of measurement error, e.g. a lower ICC value tends to occur in a homogenous study sample [19]. Thus, in this study, the ICC estimates may have been underestimated due to a homogenous and healthy study population; whereas the measurement error estimates were probably less affected.

## Conclusion

The WHO-5 Well-being Index showed acceptable test-retest reliability in a Danish epilepsy outpatient population, but the measurement error of the scale was relatively large. Different methods of administration did not influence the results. Further studies are required to provide insight into the test-retest reliability and measurement error in different language versions of the WHO-5 Well-being Index and in different patient populations.

## Abbreviations

CI: Confidence Interval
ICC: Intraclass correlation coefficients
IQR: Inter quartile range
LOA: Limits of agreement
MDC: Minimal detectable change
PRO: Patient-reported outcome
SD: Standard deviation
SEM: Standard error of the measurement
WHO-5: WHO-5 Well-being Index
